# Recommended irrigation volume for an intravenous port: Ex vivo simulation study

**DOI:** 10.1371/journal.pone.0201785

**Published:** 2018-08-14

**Authors:** Ching-Yang Wu, Chia-Hui Cheng, Jui-Ying Fu, Yen Chu, Ching-Feng Wu, Chien-Hung Chiu, Po-Jen Ko, Yun-Hen Liu

**Affiliations:** 1 College of Medicine, Chang Gung University, Taoyuan, Taiwan; 2 Division of Thoracic and Cardiovascular Surgery, Department of Surgery, Chang Gung Memorial Hospital at Linkou, Taoyuan, Taiwan; 3 Laboratory of Cardiovascular Physiology, Department of Medical Research and Development, Chang Gung Memorial Hospital at Linkou, Taoyuan, Taiwan; 4 Chest Division, Department of Internal Medicine, Chang Gung Memorial Hospital at Linkou, Taoyuan, Taiwan; University of Utah Hospital, UNITED STATES

## Abstract

**Background:**

An intravenous port, which differs from a central venous catheter, has an injection chamber at the end of the catheter. This structural difference causes the irrigation flow pattern to be quite different from that of the central venous catheter. Furthermore, the intraluminal volume differs due to the size of the injection chamber and implanted catheter length. Hence, the ideal recommended irrigation volume varies because of differences in intraluminal volume, however, the recommended irrigation volume is 10 ml and may be a cause for reported port malfunctions. This study investigates the best irrigation volume for an intravenous port by simulating the clinical scenario ex-vivo to access its usefulness.

**Materials and methods:**

This study was composed of two tests. The irrigation volume test attempted to quantify the irrigation volume of an implanted port while the irrigation rate test attempted to simulate daily nursing practice in order to clarify the effect of irrigation flow. The human blood needed for the simulation was donated by volunteers and the total volume was 10 ml per test. The irrigation volume test was done by syringe pump with varying pre-set irrigation volume after the port and connected catheter were filled with volunteer blood. After irrigation with pre-set volume, the retained intraluminal solution was collected and quantified by Bradford assay in order to titrate the best irrigation volume. The irrigation rate test tried to simulate daily maintenance practice in different settings with the quantified irrigation volume as identified by the irrigation volume test. The retained intraluminal solution was collected and quantified by Bradford assay in order to confirm the efficacy of the quantified irrigation volume.

**Results:**

In both SVC and IVC ports, we identified the twenty times the intravascular volume as sufficient for a complete wash out of the blood component in the irrigation volume test. The minimal irrigation volume for SVC and IVC port were 10 ml and 15.6 ml respectively. In irrigation rate test, the irrigation for SVC and IVC port was 10 and 20 ml, respectively, for the sake of preparation convenience. We not only identified the importance of preparation, i.e. irrigation of the extension line but also confirmed the efficacy of the recommended irrigation volume.

**Conclusion:**

The irrigation volume should be varied according to the intraluminal volume. Maintenance should be performed after the extension line has been irrigated. The recommended port irrigation volume for SVC and IVC route were 10 and 20 ml, respectively.

## Introduction

An intravascular port can provide secure vascular access for use in anti-cancer therapy. Good maintenance is crucial to keep the port functional. Though proper maintenance, any retained blood component and residual drug precipitate can be completely washed out, resulting in a clear catheter lumen and injection chamber. Irrigation volume and irrigation flow rate are two factors that play a crucial role in port maintenance. Irrigation volume depends on the intraluminal volume, including the catheter lumen and the injection chamber of the implanted intravenous port, and varies with different manufacturers and implanted catheter length. However, published guidelines and manufactures’ maintenance manuals recommend 10 ml normal saline irrigation volume for an intravenous port, regardless of the injection chamber size and implanted catheter length.[1–4]

With regard to irrigation flow, the flow orientation and its pattern need to be considered. From the literature review, the best irrigation flow orientation is observed when the opening of the needle is completely opposite to the opening of the injection chamber[5], while the pulsatile flush technique has been reported to prevent bacterial colonization and fibrin deposit in central venous catheters.[6,7] However, these goals are difficult to achieve in actual port maintenance. The ideal flow orientation cannot be achieved in clinical practice because the implanted port orientation cannot be directly seen. Furthermore, the pulsatile flush technique used for central venous catheter may be ineffective for intravenous port because the rush of the flow would be diminished after entering the board cylindrical injection chamber attached at the end of the catheter.

**Fig 1:**
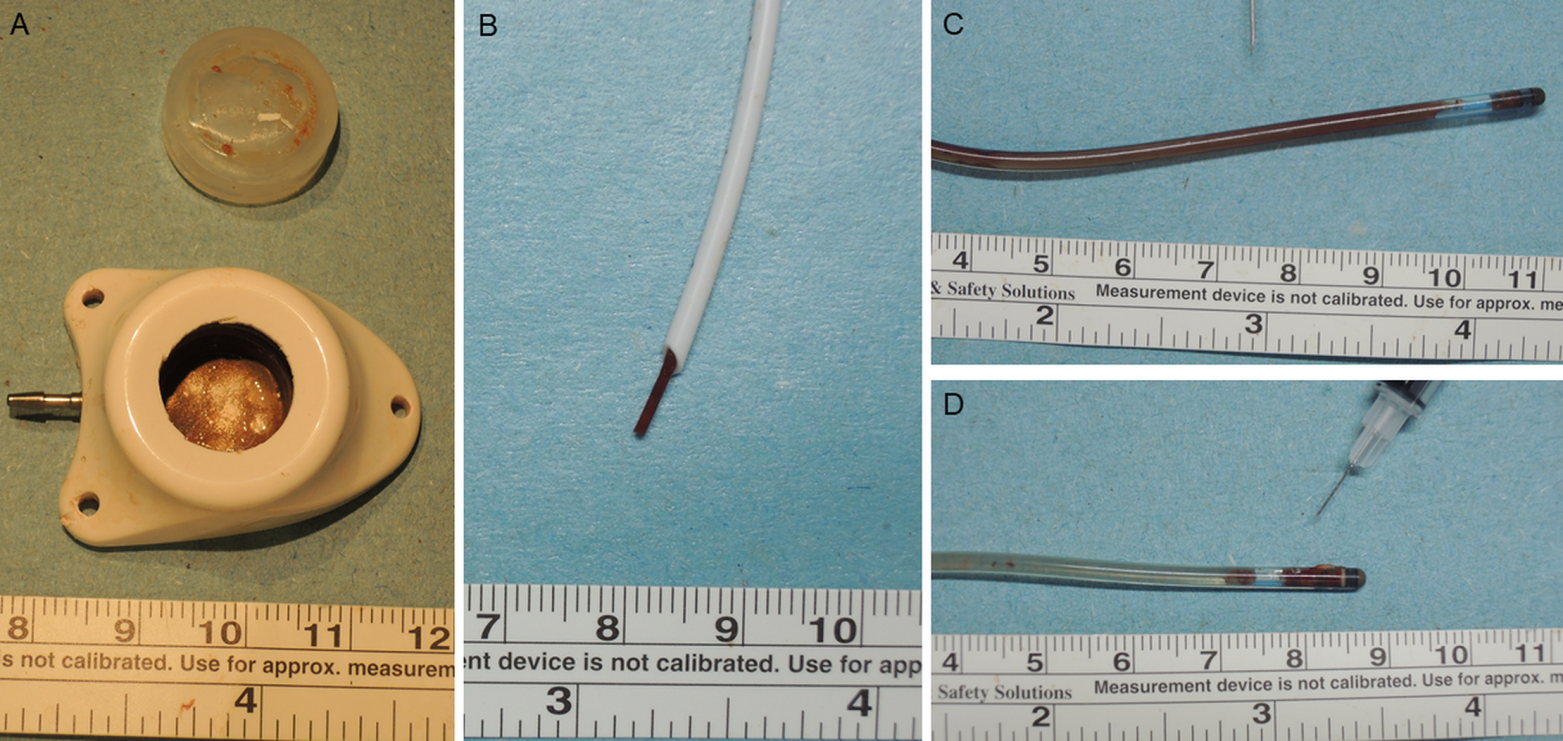
Clinical presentation of implanted intravenous port after chemotherapy. A: Fibrin deposit (Injection chamber, B’Braun Fr 6.5). B: Catheter tip clot (Open tip catheter, B’Braun Fr 6.5). C: Catheter clot (Valved tip; Bard Fr. 8). D: Catheter tip clot (Valved tip; Bard Fr. 8).

From the point of view of clinical practice, we found that fibrin deposit, and blood clots remaining in the catheter and catheter tip are common (Fig 1) in implanted intravenous ports under current maintenance strategies, ie. when irrigated with 10 ml normal saline, followed by heparin solution as a catheter lock. In addition, the reported malfunction rate of an intravenous port in our institute varied from 0.99~2.98%.[8,9] The reported incidence of intravenous port malfunction varied from 0.0051 to 0.0772 episode per 1000 catheter-days.[8.9] Based on this evidence, the current recommended irrigation volume appears insufficient for port maintenance. The literature review failed to provide any clearly quantified recommended irrigation volume for different clinical scenarios, leaving unanswered the question of whether current irrigation volume is sufficient for port maintenance. We designed an ex vivo simulation study to cover different scenarios in order to quantify sufficient irrigation volume.

## Materials and methods

### Study design

This study was an ex-vivo study and consisting of two tests, including irrigation volume and irrigation rate test. The irrigation volume test attempted to quantify the irrigation volume of an implanted port and irrigation flow test attempted to simulate daily nursing practice in order to clarify the effect of irrigation flow. Informed consent was given to volunteer and blood sampling was done after volunteer agreed and signed the inform consent. The human blood needed for the simulation was donated by volunteers and total volume was 10 ml per test. This study was approved by the Chang Gung Memorial Hospital Institutional Review Board (101-4798A3). All methods were carried out in accordance with approved guidelines.

### Port selection and choice of catheter length

We chose Bard Fr 6.6 port (Bard Access Systems Inc, Salt Lake City, UT, USA) as our test subject. The volumes of the injection chamber and catheter were 0.3 ml and 0.008 ml/cm, respectively.[10] For ports implanted via the superior vena cava (SVC), the implanted catheter length was determined using associated Height/10 cm[11,12], whereby additional catheter length was needed to run parallel to the axis of the SVC in order to avoid catheter impingement. For ports implanted via the inferior vena cava (IVC), the catheter was implanted via the greater saphenous vein (GSV), and iliac vein to the junction site between IVC and right atrium. Additional subcutaneous tunnel was needed from the GSV to the anterior inferior iliac spine. From our implantation experience, the total catheter length was 50–60 cm. Therefore, we chose 25 and 60 cm as tested catheter length for SVC and IVC implantation, respectively.

### Blood donation and preparation

The blood utilized in the simulation test was donated by the volunteer just prior to simulation. Informed consent was obtained prior to donation. The donated blood was filled into the new attached port with selected catheter length via a non-coring puncture needle. In order to simulate real clinical nursing practice, no anticoagulant agent was added. Total donated blood volume in each simulation was 10 ml, all of which was used for the simulation.

### Equipment and simulation set up

**Fig 2:**
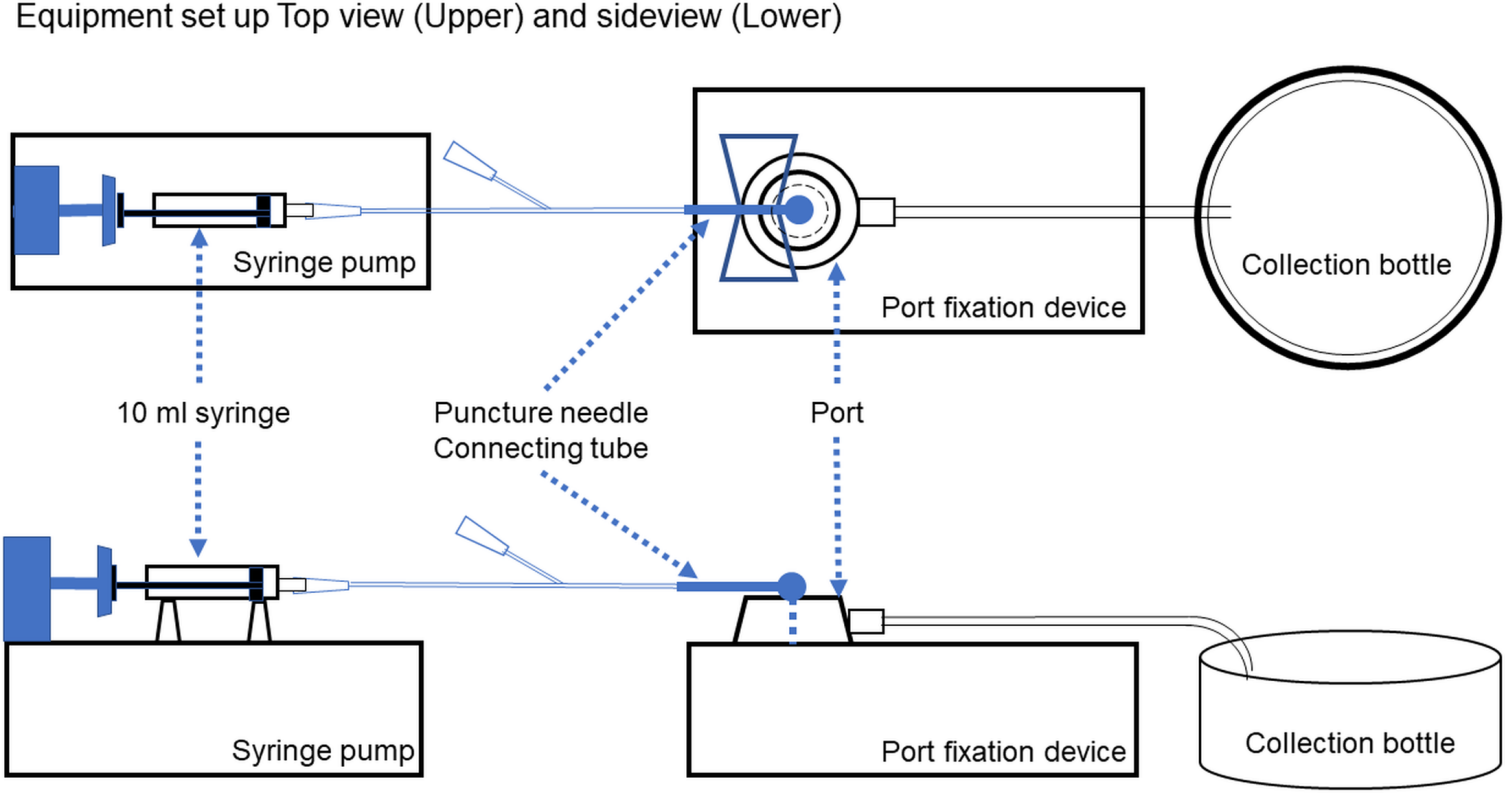
Equipment and simulation set up.

The standard puncture site was the center of the silicone diaphragm and the non-coring puncture needle was inserted perpendicular to the silicone diaphragm. The opening was placed diametrically opposite to the direction of the opening of the injection chamber as suggested by Guiffant G et al.[5] Two different types of puncture needles were used for cannulation, one being a 20G metallic coring only (Bard Access Systems Inc, Salt Lake City, UT, USA) and the other a 20 G puncture needle (Bard Access Systems Inc, Salt Lake City, UT, USA) Fresh human blood was donated by healthy young male volunteers just before the irrigation simulation. We utilized Harvard PHD 2000 Syringe Pump (Instech Laboratories Inc, Montgomery, PA, USA) for the selected injection rate. (Fig 2)

### Quantification of protein deposit

We used 0.05% trypsin (Gibco, Grand Island, NY, USA) to digest the cellular content within the catheter. The cellular protein levels were quantified by Bradford assay (BioRad, Hercules, CA). Briefly, 5 standard dilutions of a bovine serum albumin with a range of 5 to 100 μg of protein were prepared and sample solutions were assayed in triplicate, whereby the absorbance at 595 nm was measured.

### Irrigation volume test

The test attempted to quantify the irrigation volume needed for adequate irrigation. A new empty port with selected catheter length was assembled first. A non-coring puncture needle with or without connecting tube was inserted at the center of the silicone diaphragm with orientation opposite to the opening of the injection chamber. The port and its attached catheter were filled with volunteer blood using the puncture needle. After preparation, the puncture needle was removed and a new non-coring puncture needle was re-inserted at the same site of the silicone diaphragm with the same orientation. Normal saline irrigation was conducted via the puncture needle with the aid of a syringe pump, with the irrigation volume set at 1-times, 2-times, 5-times, and 20-times the intravascular volume. In order to simulate actual clinical use, another five simulations were performed via puncture needle with connecting tube (Table 1) In order to simulate irrigation after implantation, five simulations were done via puncture needle without connecting tube. (Table 1) The infusion rate was set at 38 ml/ min, ie 0.63 ml/sec, which is similar to daily clinical use. After irrigation was complete, the entire retained solution in the port and catheter was collected and quantified by Bradford assay (BioRad, Hercules, CA)

**Table 1:** Irrigation volume test.

Bard Port Fr.6.6, Catheter length 25cm, Total volume: 0.5 ml											
Volume multiples	Irrigation volume (ml)	Puncture needle (with connecting tube, volume 0.2 ml)					Puncture needle (without connecting tube)				
		Residual protein (μg)					Residual protein (μg)				
		1^st^	2^nd^	3^rd^	4^th^	5^th^	1^st^	2^nd^	3^rd^	4^th^	5^th^
**1x**	**0.5**	283.2	382.4	274.1	275.5	131.2	184.8	57.5	58.4	78.5	77.9
**5x**	**2.5**	5.95	19.76	19.3	6.8	6.7	14.8	5.2	0	7.4	7.7
**10x**	**5**	0	0	0.6	0	0	0	0	0	0	0
**20x**	**10**	0	0	0	0	0	0	0	0	0	0
Bard Port Fr.6.6, Catheter length 60 cm, Total volume: 0.78 ml											
Volume multiples	Irrigation volume (ml)	Puncture needle (with connecting tube, volume 0.2 ml)					Puncture needle (without connecting tube)				
		Residual protein (μg)					Residual protein (μg)				
		1^st^	2^nd^	3^rd^	4^th^	5^th^	1^st^	2^nd^	3^rd^	4^th^	5^th^
**1x**	**0.78**	266.4	374	239.4	272.2	199.3	155.4	123.8	44.4	72.5	74.3
**5x**	**3.9**	0	11.22	15.2	6.4	0	13.9	10.7	0	0	0
**10x**	**7.8**	0	0	4.2	0	0	0	1.8	0	0	0
**20x**	**15.6**	0	0	0	0	0	0	0	0	0	0

### Irrigation rate test

The goal of the test was to simulate daily maintenance practice with an ex-vivo environment in order to confirm its efficacy. A new empty port with selected catheter length was assembled first. A non-coring puncture needle with connecting tube was inserted at the center of the silicone diaphragm with orientation opposite to the opening of the injection chamber. The port was filled with volunteer blood by the withdrawal method until the connecting tube of the puncture needle was filled with blood. In Scenario1, we simulated maintenance of the implanted SVC port with puncture needle without any preparation. In Scenario 2, we simulated maintenance of the implanted SVC port with puncture needle after irrigating the connecting tube. In Scenario 3, we simulated maintenance of the implanted IVC port with puncture needle after irrigating the connecting tube. The irrigation volumes for the port with 25 cm catheter and 60 cm catheter were 10 and 20ml normal saline, respectively. Two different infusion rates were set at 38 ml/ min and 19 ml/ min, respectively. Twelve simulations of each clinical scenarios were conducted, whereby six were done by slow irrigation date, and six at a fast flow irrigation rate. After irrigation was completed, the entire retained solution in the port and catheter was collected and quantified by Bradford assay. (BioRad, Hercules, CA)

### Statistics

We used the Kruskal-Wallis test to determine the difference between each irrigation in each scenario in the irrigation volume test. A *p*-value less than 0.05 was considered statistically significant. All analyses were performed using SAS, version 9 (SAS Institute, NC, USA).

## Results

**Fig 3:**
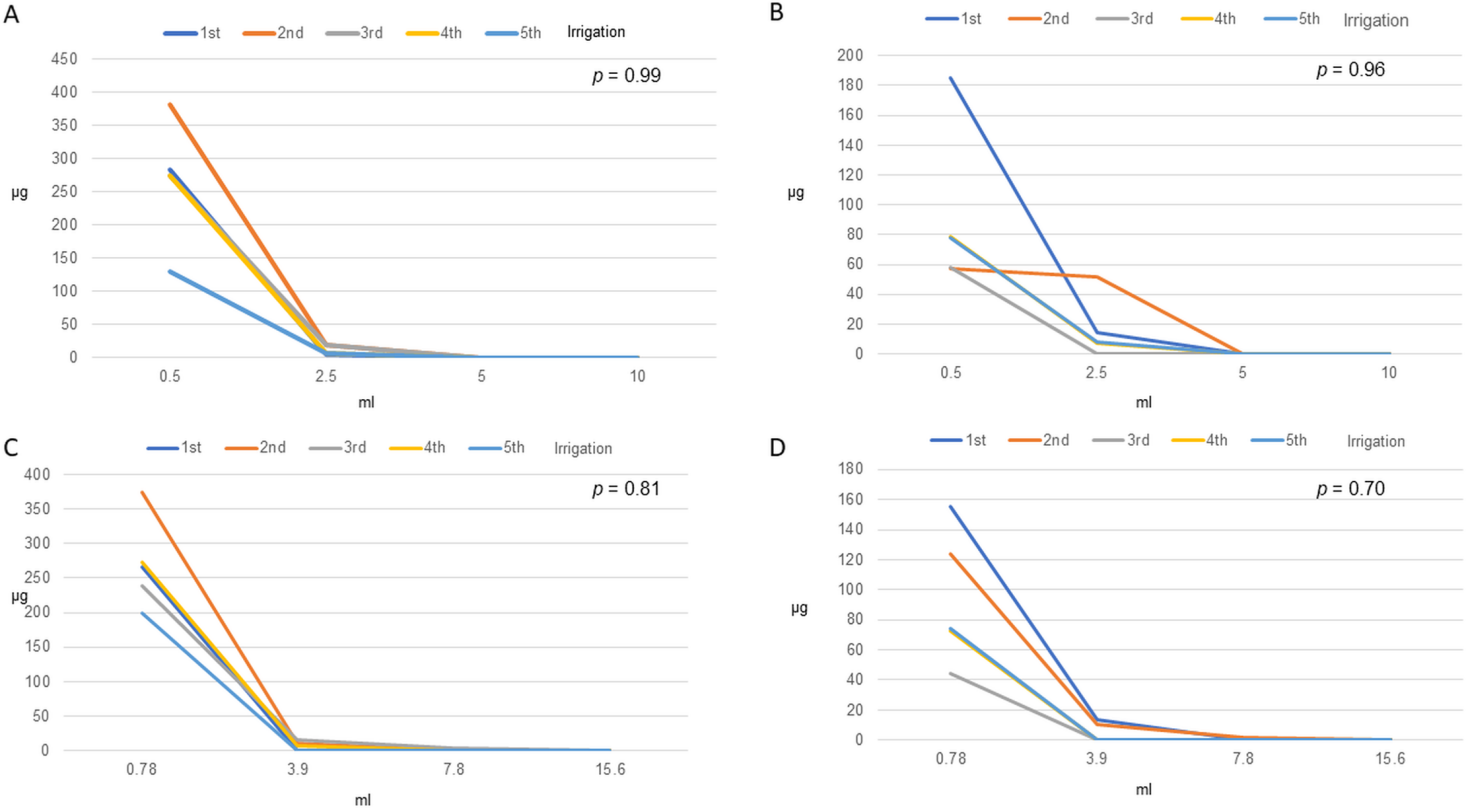
Residual protein after irrigation. A. Bard Port Fr. 6.6 (Catheter length 25 cm, Total volume: 0.5 ml) + Puncture needle (with connecting tube, volume 0.2 ml). B. Bard Port Fr. 6.6 (Catheter length 25 cm, Total volume: 0.5 ml) + Puncture needle (without connecting tube). C. Bard Port Fr. 6.6 (Catheter length 60 cm, Total volume: 0.78ml) + Puncture needle (with connecting tube, volume 0.2 ml). D. Bard Port Fr. 6.6 (Catheter length 60 cm, Total volume: 0.78 ml) + Puncture needle (without connecting tube).

In the irrigation volume test, we tried to simulate two common clinical scenarios, including SVC and IVC port. The calculated intravascular volumes of SVC port and IVC port were 0.5 and 0.78 ml, respectively. We used two types of non-coring puncture needle for irrigation volume simulation: the puncture needle with connecting tube, as commonly used in the ordinary ward, and the puncture needle without connecting tube, as commonly used in functional tests in the operation room after implantation. Prior to irrigation, fresh blood sampled from volunteers was used to fill the test port and catheter. Then the non-coring needle was inserted perpendicularly at the center of the silicone diaphragm and irrigated with normal saline solution. The tested irrigation volume was then increased in multiples of the calculated intravascular volume. For the SVC port, we were able to determine that twenty times the intravascular volume, ie. 10 ml, could completely wash out blood components, leaving no protein residue within the lumen. For the IVC port, the ideal irrigation volume was the twenty times the total volume, which was equal to 15.6 ml. (Table 1) We further compared the different irrigation curves in each scenario in the irrigation volume test, whereby our results showed no difference between irrigations, thus further confirming the reproducibility. (Fig 3)

**Fig 4:**
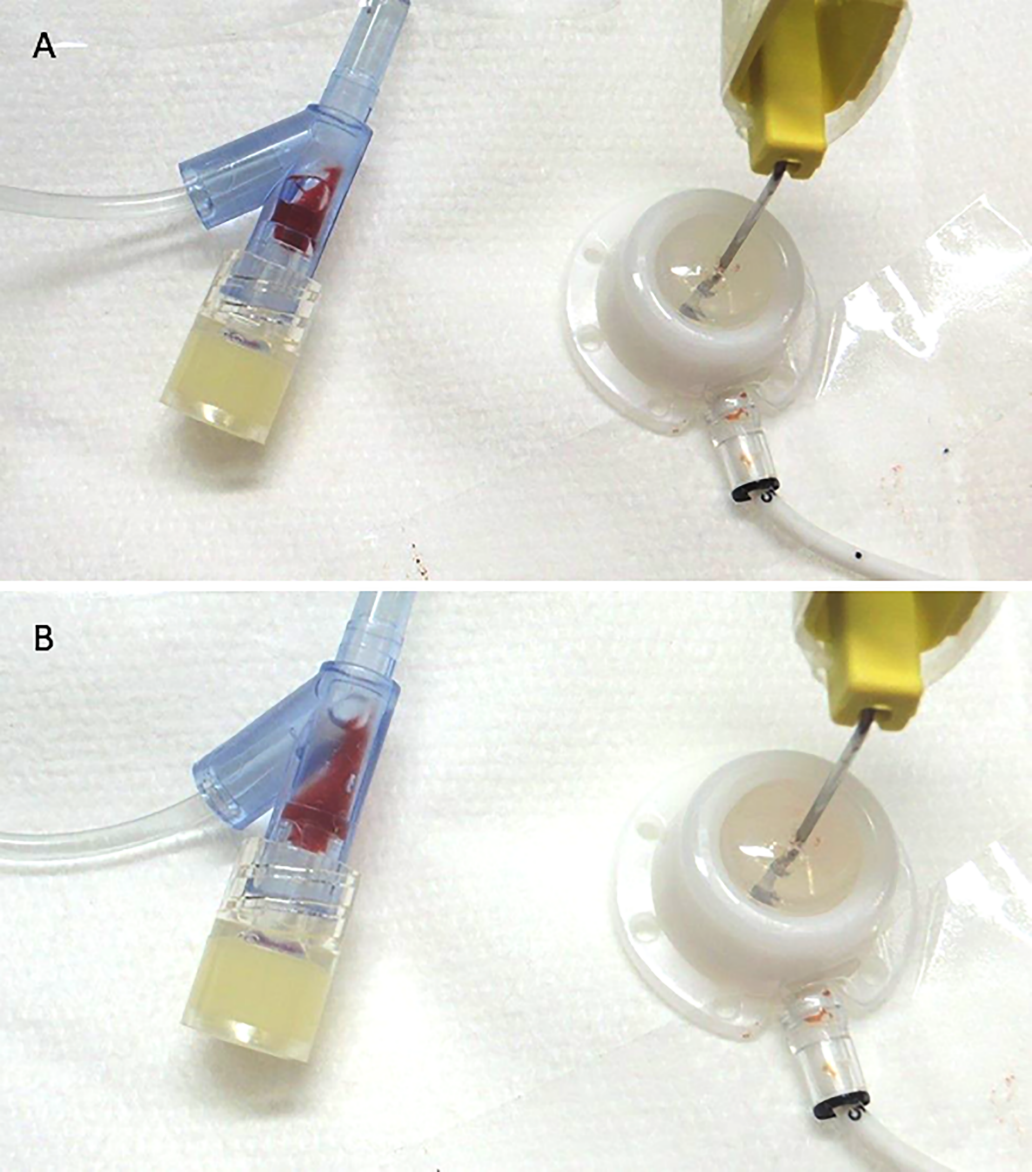
Blood retained in Y-connector of non-coring puncture needle. A. Irrigated with low flow rate (19 ml/min). B. Irrigated with fast flow rate (38 ml/min). More gross visible blood was retained in Y-connector because of the vortex.

In the irrigation rate test, we simulated three commonly encountered clinical scenarios. (Table 2) The irrigation volume was based on the irrigation volume test. In this study, 10 ml was used for SVC port irrigation and 20 ml for IVC port irrigation because these are easily prepared from commercially available packages of normal saline containing 10 ml per package. Simulation 1 represented the situation of a patient with implanted SVC port, whereby port irrigation was performed without cleaning the connecting tube of the puncture needle. Fresh blood sampled from a volunteer was used to fill the tested port and catheter, and a non-coring puncture needle was inserted perpendicularly at the center of the diaphragm. In this situation, we were able to identify a vortex at the junction between the connecting tube and the Y-connector, with blood being retained at this site under both slow and fast irrigation flow rate. (Video 1, 2) However, at the faster flow rate, the vortex increased and more blood component was retained in this area. (Fig 4) Simulations 2 and 3 represented patients with implanted SVC and IVC port, respectively, and irrigation was performed after cleaning the connecting tube of the puncture needle. In these two situations, no more retained blood was noted at the junction between the connecting tube and the Y-connector. We identified the correct irrigation volume to irrigate the port well and leave no remaining blood component and confirmed it by irrigation rate test.

**Table 2:** Irrigation rate test.

Settings	Simulation (1)		Simulation (2)		Simulation (3)	
Equipment	①Port: Bard Fr 6.6 ②Catheter length: 25cm ③Injection chamber 0.3 ml ④Catheter: 0.2 ml ^1^ ⑤Connecting tube volume: 0.2ml		①Port: Bard Fr. 6.6 ②Catheter length: 25cm ③Injection chamber 0.3 ml ④Catheter: 0.2 ml ^1^ ⑤Connecting tube volume: 0.2ml		①Port: Bard Fr. 6.6 ②Catheter length: 60 cm ③Injection chamber 0.3 ml ④Catheter: 0.48 ml ^2^ ⑤Connecting tube volume: 0.2ml	
Flush volume	10 ml		10 ml		20 ml	
Pre-irrigation preparation	None		Connecting tube of puncture needle had been irrigated and no gross blood was identified in Y-connector.		Connecting tube of puncture needle had been irrigated and no gross blood was identified in Y-connector.	
	Rate					
Protein (μg)	0.63ml/sec	0.32ml/sec	0.63ml/sec	0.32ml/sec	0.63ml/sec	0.32ml/sec
1^st^	0.0	0.0	0.0	0.0	0.0	0.0
2^nd^	0.0	0.0	0.0	0.0	0.0	0.0
3^rd^	***1*.*2***	0.0	0.0	0.0	0.0	0.0
4^th^	***0*.*3***	0.0	0.0	0.0	0.0	0.0
5^th^	0.0	0.0	0.0	0.0	0.0	0.0
6^th^	0.0	0.0	0.0	0.0	0.0	0.0

^1.^ Intra-catheter volume for port via superior vena cava: 0.008 ml/ cm * 25 cm catheter^¶^

^2.^ Intra-catheter volume for port via inferior vena cava: 0.008 ml/ cm * 60 cm catheter^¶^

## Discussion

Totally implantable intravenous ports were first designed and utilized for clinical use by Dr. Niederhuber in 1982.[13] They provide secure vascular access and decrease the frequency of venipuncture. However, the reported malfunction rate of intravenous ports has varied from 0.87 to 12.5%.[14–19] There are many factors, including implantation and maintenance issues related to port malfunction. Our previous study found that inadequate pocket creation could cause surrounding subcutaneous tissue to push the port body upward, causing catheter impingement.[20] This, in turn, could compress the catheter lumen and lead to inadequate irrigation, resulting in malfunction. After we proposed a standard algorithm for intravenous port implantation, there were no procedure related complications but the malfunction rate remained at 0.99%.[9] This result suggests that maintenance plays a role in port malfunction. In addition, photographic evidence from removed ports shows fibrin deposit and blood clot accumulation are not uncommon. (Fig 1) This evidence implies that current maintenance strategies may be inadequate and in need of modification.

The crucial factors in of port maintenance include irrigation orientation, irrigation method, and irrigation volume. With regard to irrigation orientation, Guiffant G et al recommended that the puncture needle should be precisely in the center of the diaphragm and the opening of the puncture needle should be opposite to the opening of the injection chamber, as referenced by chest plain film.[5] However, the silicon diaphragm and opening of the injection chamber are invisible, preventing nursing staff from performing ideal puncture. Nursing staff need to be able to identify the center of the implanted port and puncture with a non-coring needle perpendicular to the port. As regards irrigation volume, this is correlated to intraluminal volume and irrigation flow pattern. The flow pattern of an intravenous port is quite different from a central venous catheter, having a vortex within the injection chamber and laminal flow in the attached catheter. In addition, the luminal volume varies between different manufacturers. However, the recommended irrigation volume for an intravenous port according to the instruction manual and guidelines is 10 ml regardless of the intraluminal volume variations.[1–4] Therefore, we tried to quantify a recommended irrigation volume by the irrigation volume test. In our study, the ideal irrigation volume was found to be 20 times the intra-luminal volume of the implanted port, including the injection chamber and catheter lumen. For an SVC port, 10 ml normal saline was recommended for irrigation if the implanted catheter length was less than 25 cm. For an IVC port, 20 ml normal saline was recommended for irrigation if the implanted catheter length was less than 60 cm. Our result provides quantified irrigation volume data and shows that the ideal irrigation volume is more than in previously recommended guidelines.[1.4] From the point of view of irrigation method, pulsatile flush technique could result in unsteady flow in the central venous catheter, reducing the time scale for de-adhesion of solid deposits compared with laminar flow.[6.7] However, this technique may not be suitable for intravenous port because of the difference in structure. The intermittent rush of flow cannot create a continuous vortex within the injection chamber and may lead to inadequate irrigation. Therefore, we used a continuous flush technique in this study. We also clarified the importance of connecting tube irrigation. Simulation 1, the possibility of inadequate irrigation remained because blood might retain at the junction site of the Y-connector. In the irrigation flow rate test, we confirmed the irrigation efficacy of the recommended irrigation volume and connecting tube preparation.

According to our findings, the main concepts for port maintenance strategy are as follows. First, puncture the port with a non-coring needle in a direction perpendicular to the body surface in order to minimize the gap, by ideal puncture. Second, irrigate the connecting tube and Y-connector until no gross blood can be seen. Third, irrigate the intravenous port with the recommended irrigation volume as follows: 10 ml normal saline for a port implanted via the superior vena cava route and 20 ml for a port implanted via the inferior vena cava route. Fourth, complete catheter lock procedure. However, further investigation is warranted to confirm the clinical efficacy of aforementioned strategy. Some limitations apply to our study. First, differences among manufacturers could lead to variation of intravascular volumes, necessitating different irrigation volumes for different manufacturers’ products. The precise irrigation volume can be calculated from intraluminal volume as documented in the instruction manuals and determined by irrigation volume test. Second, this study was a study with small samples. However, the commercially available products come with uniform minimal differences, which could render a test with test with large sample size unnecessary. Third, we designed an ex-vivo simulation of the current maintenance strategy was conduct under laboratory conditions and further clinical investigation is warranted. Despite these limitations, our study not only provides precise irrigation volumes for implanted ports but also shows the importance of preparing the connecting line, and thus contributes to basic knowledge on standard port maintenance.

## Conclusion

The irrigation volume should be varied according to intraluminal volume. Maintenance should be performed after the extension line has been irrigated. The recommended port irrigation volume for SVC and IVC route was 10 and 20 ml, respectively.

## Supporting information

S1 Movie Port irrigation slow.MOV. (MOV)

S2 Movie Port irrigation fast.MOV. (MOV)
